# Night-shift work duration and breast cancer risk: an updated systematic review and meta-analysis

**DOI:** 10.1186/s12905-021-01233-4

**Published:** 2021-03-02

**Authors:** E. Manouchehri, A. Taghipour, V. Ghavami, A. Ebadi, F. Homaei, R. Latifnejad Roudsari

**Affiliations:** 1 Department of Midwifery, School of Nursing and Midwifery, Mashhad University of Medical Sciences, Mashhad, Islamic Republic of Iran; 2 Department of Epidemiology, School of Health, Mashhad University of Medical Sciences, Mashhad, Islamic Republic of Iran; 3 Department of Biostatistics, School of Health, Mashhad University of Medical Sciences, Mashhad, Islamic Republic of Iran; 4 Behavioral Sciences Research Center, Lifestyle Institute, Baqiyatallah University of Medical Sciences, Tehran, Islamic Republic of Iran; 5 Nursing Faculty, Baqiyatallah University of Medical Sciences, Tehran, Islamic Republic of Iran; 6 Cancer Research Center, Faculty of Medicine, Mashhad University of Medical Sciences, Mashhad, Islamic Republic of Iran; 7 Nursing and Midwifery Care Research Center, Mashhad University of Medical Sciences, Mashhad, Islamic Republic of Iran

**Keywords:** Breast cancer, Meta-analysis, Night-shift work, Shift-work, Systematic review

## Abstract

**Background:**

The International Agency for Research on Cancer (IARC) has classified shift work as a possible human carcinogen. The results of systematic on this topic is contradictory. This systematic review and meta-analysis, therefore, aimed to update the current evidence regarding the relationship between night-shift work duration and breast cancer risk.

**Methods:**

PubMed, Web of Science, and Scopus as well as reference list of included studies were searched until December 19, 2020. Observational case–control or cohort studies investigating the relationship between the duration of night-shift work and breast cancer in women were included, which all quantified night-shift work exposure. All statistical analyses were done by Stata version 11.2.

**Results:**

Our literature search was resulted in retrieval of 4854 publications from which 26 eligible studies with 1,313,348 participants were included in the meta-analyses. The pooled relative risk (RR) and 95% confidence intervals (CIs) of breast cancer for short-term night-shift workers (< 10 years) was 1.13 (95% CI 1.03–1.24, p = 0.008, I^2^ = 71.3%), and for long-term night-shift workers (≥ 10 years) was 1.08 (95% CI 0.99–1.17, p = 0.09, I^2^ = 42.2%), with moderate to substantial statistical heterogeneity observed in both analyses. The results of subgroup analysis showed that flight attendants with long overnight flights were at an elevated risk of breast cancer, but unmeasured confounders limited these results. The risk of breast cancer in case control studies, adjusted for reproductive factors and family history of breast cancer as well as studies with high quality was increased in both short term and long term night-shift workers.

**Conclusions:**

This systematic review found a positive statistical relationship between night work and breast cancer risk in short-term night-shift workers but no increase was observed in the long-term night-shift workers.

## Background

The most widely identified invasive cancer among women is breast cancer (BC), afflicting one in eight women [1]. 25% of all cancers and 15% of deaths in women are related to BC [2]. Not all women run the same risk of BC during their lives, but specific factors, called risk factors, increase their chances of contracting the disease. The proportion of diseases that could be prevented by decreased exposure to modifiable risk factors, can help to recognize where and for whom such preventative disease‐specific strategies need to be dedicated [3]. Research found that around half of the global cancer burden is the consequence of some modifiable factors including diet, obesity, sedentary lifestyle as well as endocrine disrupting chemicals and can thus be prevented [4].

Khakbazan et al. [5] reported that the increase in life expectancy along with the propensity to adopt a western lifestyle has changed BC into a growing public health concern in many developing countries.

An issue taken into account in numerous epidemiological studies over the past decade is the effect of shifts on BC [6]. Shift work refers to a work schedule that is outside the standard 9 am to 5 pm, including evening or night shifts, early morning shifts, and rotating shifts [7]. According to a 2009 IARC working group, a night-shift is described as ≥ 3 h of work between midnight and 5 am [8].

Shift work exists in many industries and factories such as the oil industry, power plants, and iron and steel industries, fields related to medicine, midwifery, and nursing, and fire department, law enforcement, and water, electricity, and telephone services [9]. This type of work can lead to disturbed circadian rhythm, diminished melatonin hormone, and sleep disturbances that affect hundreds of metabolic and physiological processes, including synthesis of hormones, apoptosis, and cell cycle life and trigger tumors such as BC in the human body [10, 11]. According to the data collected in 2015, in the sixth EU Survey on Working Conditions, 14% of the female working population reported working during the night [12].

The disruption in the circadian rhythm following exposure to light at night (LAN) has long been considered as a possible cause of BC [13, 14]. The shift work, in addition to exposure to LAN, results in irregularities in eating as well as social and familial relationships [15]. Possible mechanisms for carcinogenesis of LAN are suppressed melatonin hormone, reduced immune system following sleep disorder, confusion in the body circadian system, and irregularity in cell proliferation [16].

In 2019, the IARC re-assessed night-shift work (NSW) and described it as a "probable" carcinogen (IARC Group 2A) [17]. However, the results of the systematic reviews and meta-analyses on the relationship between night-shift work and BC have been contradictory [18, 19] and articles are increasingly being published with opposing results in this regard [20–22]. According to a meta-analysis on cohort studies (2015), rotating NSW increased the incidence of BC by 8.9%, and a positive dose–response relationship was found between NSW and breast tumor incidence [23]. It was found by Wang et al. in a meta-analysis that the risk of female BC would increase by 3% following each 5-year increase in NSW exposure [24], which is supported by some other meta-analyses [25–27]. But in other systematic reviews and meta-analyses this relationship has not been reported [19, 28–30]. Kamdar et al. in a meta-analysis of 15 observational studies reported weak evidence to support the association between NSW with increased BC risk [29].

Moreover, the duration of NSW has not been considered by some meta-analyses [31]. Due to the growing worldwide prevalence of shift work, the great economic burden of BC, and the large number of articles with inconsistent results, the present review aimed to investigate the relationship of night-shift work and its duration with BC risk through a systematic review and meta-analysis of the existing observational studies. In addition, in the present study, the subgroup analysis for selected variables, and a review of past meta-analyses is carried out.

## Methods

### Search strategy

Preferred Reporting Items for Systematic Reviews and Meta-Analyses (PRISMA) guidelines were adopted for reporting this systematic review and meta-analysis. PubMed, Web of Science, Google Scholar, and Scopus were browsed up to December 19, 2020. There was no limit on the initial date applied. The key words employed to identify the studies were: "shift work" OR "night work" OR "night-shift work" OR "rotating-shift work" AND "breast cancer" OR "breast carcinoma" OR "breast neoplasm". Boolean operators (AND, OR), truncation, and MeSH terminology were used appropriately for the systematic identification of data (Table 1).

**Table 1:** Strategy for systematic searches of the published literature

Search	Most recent queries
#1	Search "Breast cancer"[All Fields] OR "Breast neoplasm"[MeSH Terms] OR "breast carcinoma"[All Fields] OR "breast tumor"[All Fields]"
#2	"night shift work"[All Fields]) OR "night work"[All Fields] OR "shift work"[All Fields])
#3	#1 AND #2
#4	#3 AND published up to December 19, 2020

### Inclusion and exclusion criteria

Studies were included in the review if they had the following criteria (1) were peer-reviewed case–control, observational nested case–control, or cohort studies (2) quantified NSW in all job categories including work on domestic and/or intercontinental overnight flights), (3) provided risk ratios, odds ratios, hazard ratios, and 95% CIs for BC incidence confirmed by histopathology or through data available from Cancer Registry in females aged at least 18 years old. There was no restriction regarding country, race, publication language, and date. Excluded studies were (1) studies that reported the duration of night-shift work as ‘‘ever vs never’’ (2) those involving nighttime light exposure that was involuntary or non-work related, sleep duration, or subjects included with recurrent BC, and (3) studies that their full texts were not accessible. Identified studies using the Endnote X8.1 software were retrieved and managed.

### Study selection

At first, the titles and abstracts and then, the full texts of the studies were reviewed, separately, by two authors, and any inconsistency was discussed by a third author. There was only one case of disagreement regarding the inclusion of articles with the same population (entry of the most recent articles or articles with a larger population). Hand searching was carried out to identify further relevant studies.

### Outcome variable

The outcome variable of this study was breast cancer, which was defined as having positive diagnosis of BC based on the medical records or through data available from cancer registry.

### Quality (risk of bias) assessment

Two researchers (EM and an assistant) independently evaluated the methodological quality of individual studies using the Newcastle–Ottawa Quality Assessment Scale. Newcastle–Ottawa Scale (NOS) was examined for Cohort and case–control studies in terms of interrater reliability and construct validity in a previous study which reported a high degree of agreement across its domains [32]. The star system with a maximum of nine stars (scoring 0 to 9) was adopted by NOS, which was categorized into three parts: participant selection, comparability of study groups, exposure assessment/outcome evaluation [33]. The stars were classified as follows: 7–9 stars showed high quality, 4–6 stars meant a medium quality and 0–3 stars indicated a low quality [34].

### Data extraction

Relevant variables included the first author's name, year of publication, geographic location of participants, type of study (nested case–control, case–control or cohort studies), occupation of participants, years of follow up, source of data about outcome and exposure, definition of exposure, number of BC cases and controls (for case–control studies), cohort size (for cohort studies), risk estimates and 95% CIs for BC incidence and nightshift work duration category, source of funding and confounders for which risk estimates were adjusted. Data extraction was done separately by two researchers (EM and an assistant) using duplicate spreadsheets for validating the data extraction process. "Night shift work" was the main exposure variable, and the absence of night work was the preferred control group.

### Quantification and categorization of NSW

The duration of NSW exposure was reported in the studies included in this systematic review as an open (≥ 30 years) or closed (15–29 years) time periods. Using the midpoint for closed time periods and the minimum points for open time periods, single numeric values was assigned to each one. According to the assumption that a longer duration of NSW may be correlated with a higher incidence of BC, NSW exposure was divided into two groups: short-term (< 10 years) and long-term (> 10 years) NSW. After rounding the median of all allocated range values, the cutoff point between short-term and long-term NSW of 10 years was obtained [29].

### Subgroup analyses

Subgroup analyses were carried out by study type (nested case–control, case–control or cohort), occupation (nurses, flight attendants, or others), geographical area (Europe, North America, Asia and, Oceania), adjustment of studies for reproductive factors (yes or no), as well as a family history of BC (yes, no), quality category (high or moderate) and reporting the source of funding (yes or no) regarding short-term and long-term exposures separately.

### Statistical methods

To test the relationship between NSW and BC, risk ratios (RRs) were used. Adjusted risk assessments were preferred over crude measures where available. Then, pooled risk estimates were determined using random-effect models for the short-term and long-term NSW groups that were used due to substantial heterogeneity (p < 0.05) present in some studies. In studies that have reported multiple RRs for NSW duration, if there was more than one stratification in each short- or long-term category, we used the method of combining effect sizes across multiple comparisons within individual studies introduced by Borenstein et al. [35]. Using the × 2 and I2 statistics, statistical heterogeneity between studies was assessed. Heterogeneity was considered to be high if the I^2^ statistic was greater than 50% [36]. In order to further investigate the risk ratio in the study population, subgroup analysis was performed. We used funnel plots, Egger [37] and Begg [38] tests for assessing the publication bias. Statistical significance was considered as p < 0.05. All analyses were conducted using Stata version 11.2 (StataCorp, College Station, Texas).

## Results

### Selection of studies

**Fig. 1:**
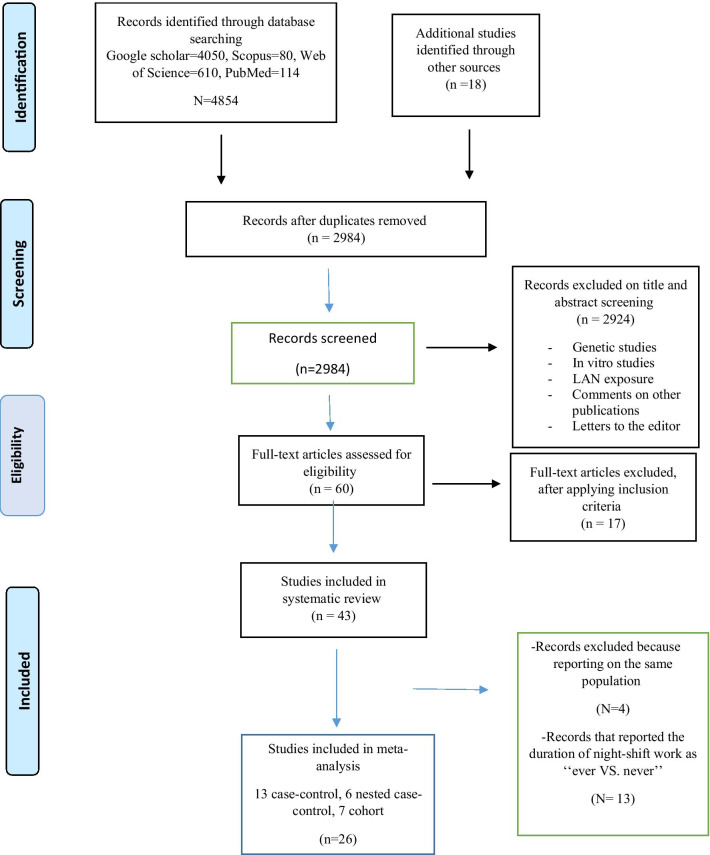
PRISMA Flowchart of the study selection process

Details of the literature review and study selection process are shown in Fig. 1. The search yielded 4872 articles, of which 1888 were duplicate records. We included all the articles in previous meta-analyses [18, 25, 29] in the present study. After review of abstracts 2924 studies were excluded for the following reasons: being focused on genetic issues, being conducted as In vitro study, and reporting LAN exposure. Also, comments on other publications and letters to the editor were excluded. We reviewed 60 full-text articles, of which 17 were excluded from the study because they did not meet the inclusion criteria, while 43 satisfied all the inclusion criteria. Regarding cohorts with several reports, we used data from the publication with the longest follow-up, hence the exclusion of four articles [6, 39–41]. 13 studies reported the duration of NSW exposure as ‘‘ever’’ also were excluded [42–54]. The present meta-analysis included 26 studies: six nested case–control [20, 21, 55–58] (Table 2), 13 case–control [16, 22, 59–69] (Table 2) and seven cohort [28, 70–75] studies (Table 3).

**Table 2:** Characteristics of the selected (nested) case–control studies on breast cancer and night-shift work

Author	Sample size Case/control	Exposure data source	Adjusted confounders	Definition of night shift	NSW category	Quality score
Hansen and Lassen [20]	218/899	Nationwide pension fund and Danish military’s company registration	HRT, number of childbirths, age at menarche, length of education, occasional sun-bathing, tobacco smoking status	Working during hours beginning after 17:00 and ending before 9:00 for at least 1 year, not including overtime	Short/long	High
Hansen and Stevens [58]	310/1240	Danish nurses association	Age, family history of BC, MHT, number of mammograms	Graveyard shifts which worked after midnight (about 8 h of work between 19 and 9) for at least 1 year	Short/long	High
Li et al. [21]	1709/4780	Factory records (80%), interviews (20%)	Parity, live births, BF, alcohol	Working at least once per week for at least 6 months between midnight and 6 a.m	Short/long	Moderate
Tynes et al. [55]	50/259	Telecom registry	Duration of employment, age, and age of first birth	Years on ships known to involve night shift work and cross time zones	Short/long	Moderate
Linnersjo et al. [57]	16/174	Airline archives	None	––-	Short	Moderate
Rafnsson et al. [56]	35/140	Employment records	Age, age at first childbirth, live births	Employed full-time ≥ 5 years; frequent long distance flights due to remote Iceland location	Short	Moderate
Hansen [61]	6281/6024	Nationwide Pension Fund	Age, age at first and last childbirth, parity, SES	≥ 6 months employment in a trade where ≥ 60% of randomly surveyed women worked at night	Short	High
Fritschi et al. [22]	1205/1789	Western Australian (WA) Cancer Registry	Age, reproductive history, alcohol intake, smoking, PA and sleep, SES, remoteness of residence, family history of BC	Working for any number of hours between midnight and 0500 h	Short/long	High
Menegaux et al. [59]	1232/1317	French departments of ‘‘Coˆte d’Or’’ or ‘‘Ille-et-Vilaine’’	Age, age at menarche, age at first full-term pregnancy, parity, MHT, family history of BC in first-degree relatives, BMI, alcohol consumption, and tobacco consumption	Working for at least 1 h between 11:00 pm and 5:00 am during all or part of each job	Short	High
Grundy et al. [62]	1034/1179	BC Cancer Registry (Vancouver), Hotel Dieu Breast Assessment Program (Kingston)	Age, ethnicity; household income; education; menopausal status; use of fertility drugs, OC, non-steroidal anti-inflammatory drugs (NSAIDs), antidepressants and HRT; reproductive factors including ever having been pregnant, number of pregnancies, age at first birth, BF and age at first mammogram; family history of BC among first-degree relatives; lifestyle factors, including smoking status, pack-years smoking, lifetime alcohol consumption; and BMI	≥ 50% of time was reported to have been spent on evening and/or night shifts, capturing both rotating and permanent night shift schedules	Short/long	High
Pesch et al. [65]	857/892	Telephone interview	Age, family history, HRT, Mammograms	Working the fulltime period between 24:00–05:00 h	Short/long	High
O'Leary et al. [60]	576/585	In-person occupational history	Age, live births, family history, education, benign breast disease	Overnight shift which could start as early as 7:00 p.m. and continue until the following morning	Short	High
Davis et al. [63]	763/741	In-person occupational interview	Parity, family history, OC use, recent HRT	Graveyard shift which began after 7:00 PM and leaving work before 9:00 AM	Short	High
Papantoniou et al. [64]	1708/1778	MCC-Spain Study	Age, family history, education, marital status, BMI, Tobacco smoking, PA, sleep habits, diet habits, Menopausal status, Parity, age at first birth, BF, ever OC, ever hormonal therapy, past sun exposure	Night work was defined as a working schedule that involved partly or entirely working between 00:00 and 6:00 a.m. at least three nights per month. This definition included overnight, late evening (end after 00:00) and early morning (start before 6:00) shifts	Short/long	High
Santi et al. [67]	743/775	Questionnaire	Age, family history, level of education, OC use, alcohol consumption, number of births, and age of first menstruation	Nurses were classified as night-shift workers if they worked in hospitals	Short/long	High
Rabstein et al. [66]	857/892	GENICA (Gene–ENvironment Interaction and breast CAncer)	Family history of BC, HRT, number of mammograms, and estrogen receptor status	Ever having worked in night shifts for ≥ 1 year and working the fulltime period between 24:00–05:00 h	Short/long	High
Lie et al. [68]	172/474	Cancer Registry of Norway	Age at diagnosis, period of diagnosis, parity, family history of BC in mother or sister, hormonal treatment in the previous 2 years before diagnosis, and frequency of alcohol consumption at the time of diagnosis	Work between 12 pm and 6 am	Short/long	High
Truong et al. [16]	1126/1174	CECILE study	Age, study area, age at menarche, age at first full-term pregnancy, parity, MHT, BMI, alcohol consumption, and tobacco consumption	Work for at least 1 h between 1100 and 0500 h during all or part of each job period	Ever/short	High
Pham et al. [69]	1721/1721	Questionnaire and face to face interview	Age, educational level, number of pregnancies, age at birth of first child, body mass index, age at menarche, alcohol consumption, smoking, use of female hormone treatment, and family history of breast cancer in first degree relatives	Ever having worked in night shifts regularly between 9:00 pm and 8:00 am for at least 2 months in their lifetime	Short/long	High

*NSW* night shift work, *HRT* hormone replacement therapy, *BC* breast cancer, *MHT* menopausal hormone therapy, *BF* breastfeeding, *PA* physical activity, *SES* socioeconomic status, *BMI* body mass index

**Table 3:** Characteristics of the selected cohort studies on breast cancer and night-shift work

Author	Total size/cases sample size	Follow up length (years)	Exposure data source	Adjusted confounders	Definition of night shift	NSW category	Quality score (%)
Koppes [72]	285,723/2531	7	Labor Force Survey	Age, origin, children in the household, education, occupational group, contractual working hours, and job tenure	Work at nights, meaning between midnight and 6 am	Short/long	High
Pronk et al. [74]	73,049/717	9	In-person occupational history plus job exposure matrix	Age, age at first childbirth, parity, family history, education, work-related PA	Starting work after 10 PM at least 3 times a month for over 1 year	Short/long	High
Åkerstedt et al. [70]	13,656/463	12	Swedish Twin registry, with follow-up in the Swedish Cancer Registry	Age, education, parity, Tobacco use, BMI, PA, Alcohol consumption, coffee consumption, menopause status, hormone use, previous cancer, time to BC diagnosis	Working hours in nights at least now and then	Short/long	High
Wegrzyn [73]	78,516/5971	24	Nurses’ Health Study	Age, height, current BMI and BMI at age 18 years, adolescent body size, age at menarche and at first birth, parity, BF history, type of menopause, age at menopause, MHT use, duration of use of estrogen-only MHT, duration of use of combined estrogen and progesterone MHT, first-degree family history of BC, history of benign breast disease, alcohol consumption, PA level, and current mammography use	At least 3 nights/month in addition to days/evenings in that month	Short/long	High
Wegrzyn [73]	114,559/3570	24	Nurses’ Health Study II	Age, height, current BMI and BMI at age 18 years, adolescent body size, age at menarche and at first birth, parity, BF history, type of menopause, age at menopause, MHT use, duration of use of estrogen-only MHT, duration of use of combined estrogen and progesterone MHT, first-degree family history of BC, history of benign breast disease, alcohol consumption, PA level, and current mammography use	At least 3 nights/month in addition to days/evenings in that month	Short/long	High
Jones et al. [71]	102,869/2059	9.5	Generations Study	Alcohol use, parity, OC use, MHT use, and menopausal status	Any jobs that regularly involved work in the late evening or night (between 10 pm and 7 am)	Short/long	Moderate
Travis et al. [28]	522 246/ 4809	Established 1996 to 2001. Analyzed at December 2013	Million woman study	Age, SES, marital status, nulliparity, age at first birth, number of children, obesity, PA, alcohol consumption, smoking, first-degree relative with BC, OC use, MHT use, amount of sleep, take medication to sleep, more evening than morning type	Any time between midnight and 06:00 h, for at least 3 nights per month	Short/long	High
Travis et al. [28]	22,559/181	Recruited 1993–1999. Analyzed at December 2013	EPIC-Oxford study	Age, SES, marital status, nulliparity, age at first birth, number of children, obesity, PA, alcohol consumption, smoking, first-degree relative with BC, OC use, MHT use, amount of sleep, take medication to sleep, more evening than morning type	Any job lasting for at least one year, and occurring on a regular basis for at least one night per month or 12 nights per year	Ever/long	High
Sweeney et al. [75]	48,451/3191	Recruited 2003–2009. Analyzed at September 2017	Sister study cohort	Age, race/ethnicity, education, marital status and parity	1 h between 12:00 and 2:00 AM) for ≥ 2 years	Short/long	High

*NSW* night shift work, *PA* physical activity, *BMI* body mass index, *BC* breast cancer, *BF* breastfeeding, *MHT* menopausal hormone therapy, *SES* socioeconomic status, *OC* oral contraceptive

### Identification and description of studies

Overall, the nested case–control studies included a total of 3574 BC cases and 10,530 controls, the case–control studies comprised a total of 18,275 BC cases and 19,341 controls, and the cohort studies consisted of a total of 23,492 BC cases from an at-risk population of 1,261,628 individuals. The study population included individuals pulling rotating or overnight shifts, including nurses [40, 41, 58, 67, 68, 73], flight attendants [56, 57], military employees [20], textile workers [21], radio and telegraph operators [55] and women in different public and private companies [16, 22, 59–66, 69–72, 74, 75]. Geographically, 17 of the 26 studies belonged to European countries [16, 20, 28, 55–59, 61, 64–68, 70–72], five to the USA [60, 62, 63, 73, 75], three to Asia [21, 69, 74] and one to Oceania [22] (Table 4). One or more BC risk factors were adjusted in all but one study [57] (Table 2). All articles were published during 1996–2020, and most were published in 2013 [22, 59, 62, 66, 68]. All included articles were written in English and we did not find any article in another language.

**Table 4:** Pooled risk estimates for breast cancer and heterogeneity analysis from adjusted risk estimates

Factors stratified	Short-term (< 10 years) versus never night-shift work				Long-term (≥ 10 years) versus never night-shift work			
	No. of studies	RR (95% CI)	${I}^{2}$, %	P value	No. of studies	RR (95% CI)	${I}^{2}$, %	P value
All studies	26 (16, 20–22, 28, 55–75)	1.13 (1.03–1.24)	73.4	0.001	17 (20–22, 28, 55, 58, 62, 64–66, 69–75)	1.08 (0.99–1.17)	42.2	0.028
*Study type*								
Nested case–control	6 (20, 21, 55–58)	1.40 (0.9–2.19)	66.2	0.011	4 (20, 21, 55, 58)	1.5 (0.86–2.66)	84.7	0.001
Case–control	13 (16, 22, 59–69)	1.25 (1.8–1.44)	59.8	0.003	6 (22, 62, 64–66, 69)	1.22 (1.02–1.46)	0	0.819
Cohort	7 (28, 70–75)	1.02 (0.97–1.06)	0	0.636	7 (28, 70–75)	1.01 (0.95–1.07)	0	0.706
*Occupation*								
Flight attendants	2 (56, 57)	3.94 (1.42–10.91)	0	0.806	0	-	-	-
Nurses	4 (58, 67, 68, 73)	1.14 (0.99–1.3)	71.6	0.007	2 (58, 73)	1.25 (0.92–1.70)	81.6	0.004
Other	19 (16, 20–22, 50, 55, 59–66, 69–72, 74, 75)	1.09 (0.97–1.22)	68.8	0.001	14 (20–22, 28, 55, 62, 64–66, 69–72, 74, 75)	1.03 (0.95–1.11)	17.9	0.248
*Geographic area*								
Europe	17 (16, 20, 28, 55–59, 61, 64–68, 70–72)	1.19 (1–1.41)	72.9	0.001	10 (20, 28, 55, 58, 64–66, 70–72)	1.21 (1.02–1.44)	54.4	0.016
North America	5 (60, 62, 63, 73, 75)	1.04 (0.96–1.13)	39.1	0.145	3 (62, 73, 75)	1.05 (0.94–1.16)	0	0.417
Asia	3 (21, 69, 74)	1.04 (0.93–1.17)	0	0.7	3 (21, 69, 74)	0.94 (0.84–1.07)	16.2	0.303
Oceania	1 (22)	1.25 (1.01–1.55)	-	-	1 (22)	1.05 (0.78–1.41)	-	-
*Adjusted for reproductive variables*								
Yes	24 (16, 20–22, 28, 55–71, 73, 74)	1.15 (1.05–1.27)	72.6	0.001	15 (20–22, 28, 55, 58, 62, 64–66, 69–71, 73, 74)	1.1 (1–1.21)	47.9	0.015
No	2 (72, 75)	0.94 (0.75–1.17)	19	0.266	2 (72, 75)	0.95 (0.76–1.19)	0	0.899
*Adjusted for family Hx*								
Yes	18 (16, 22, 28, 58–60, 62–69, 71, 73–75)	1.11 (1.03–1.2)	48.7	0.009	12 (22, 28, 58, 62, 64–66, 69, 71, 73–75)	1.1 (1–1.2)	40.2	0.059
No	8 (20, 21, 55–57, 61, 70, 72)	1.16 (0.83–1.62)	83.5	0.001	5 (20, 21, 55, 70, 72)	1.06 (0.84–1.34)	44.1	0.128
*Adjusted for confounders*								
Yes	25 (16, 20–22, 28, 55, 57–75)	1.13 (1.03–1.23)	71.9	0.001	17 (20–22, 28, 55, 58, 62, 64–66, 69–75)	1.08 (0.99–1.17)	42.2	0.028
No	1 (57)	3.27 (0.54–19.85)	-	-	0	-	-	-
*Quality category*								
High	21 (16, 20, 22, 28, 58–70, 72–75)	1.15 (1.04–1.26)	73.8	0.001	14 (20, 22, 28, 58, 62, 64–66, 69, 70, 72–75)	1.1 (1–1.21)	41.4	0.042
Moderate	5 (21, 55–57, 71)	1.05 (0.78–1.40)	50.2	0.09	3 (21, 55, 71)	1.01 (0.82–1.24)	45.3	0.161
*Source of funding*								
Low risk	22 (16, 20–22, 28, 56–60, 62, 64–73, 75)	1.08 (1.01–1.17)	50.2	0.003	15 (20–22, 28, 58, 62, 64–66, 69–73, 75)	1.09 (1–1.2)	46	0.02
Unclear	4 (55, 61, 63, 74)	1.36 (0.97–1.91)	79.9	0.002	2 (55, 74)	1.02 (0.66–1.58)	16.7	0.273

*CI* confidence interval

P values represent heterogeneity

### Quality assessment

Twenty one studies had "high" quality assessment scores [16, 20, 22, 28, 58–70, 72–75] and 5 [21, 55–57, 71] articles had moderate quality assessment scores (Table 4). The quality of studies ranged from 6 to 8 points, with a median of 7 (Additional file 1: Appendix). The weakest part of the articles according to the NOS tool was the exposure/outcome domain. Three articles (11.5%) in the exposure/outcome domain had a high risk of bias [70, 73, 75]. The researchers gave similar scores to the articles.

### Exposure measurement

Measurement and stratification of NSW exposure duration varied substantially between the studies. Twenty six studies provided risk estimates with a median exposure duration of 5 years (IQR 4–6) falling under the short-term NSW group (< 10 years) [16, 20–22, 28, 55–75]. Seventeen studies provided risk estimates in the long-term NSW group (≥ 10 years), with a median exposure duration of 17.5 years (IQR, 15–23) [20–22, 28, 55, 58, 62, 64–66, 69–75]. We produce a single pooled short- or long-term risk estimate for the 22 studies with multiple risk estimates that fall under either the short- or long-term exposure categories [16, 20–22, 28, 55, 58–60, 62–68, 70–75]. Regarding the two studies that considered intercontinental flight hours as night time work [56, 57], we considered 5,000 flight hours equivalent to almost 5 years of NSW, based on published labor reports [76]. Also in another study, years of work on a ship and cross time zones were considered as NSW [55].

### Primary BC risk analyses

**Fig. 2:**
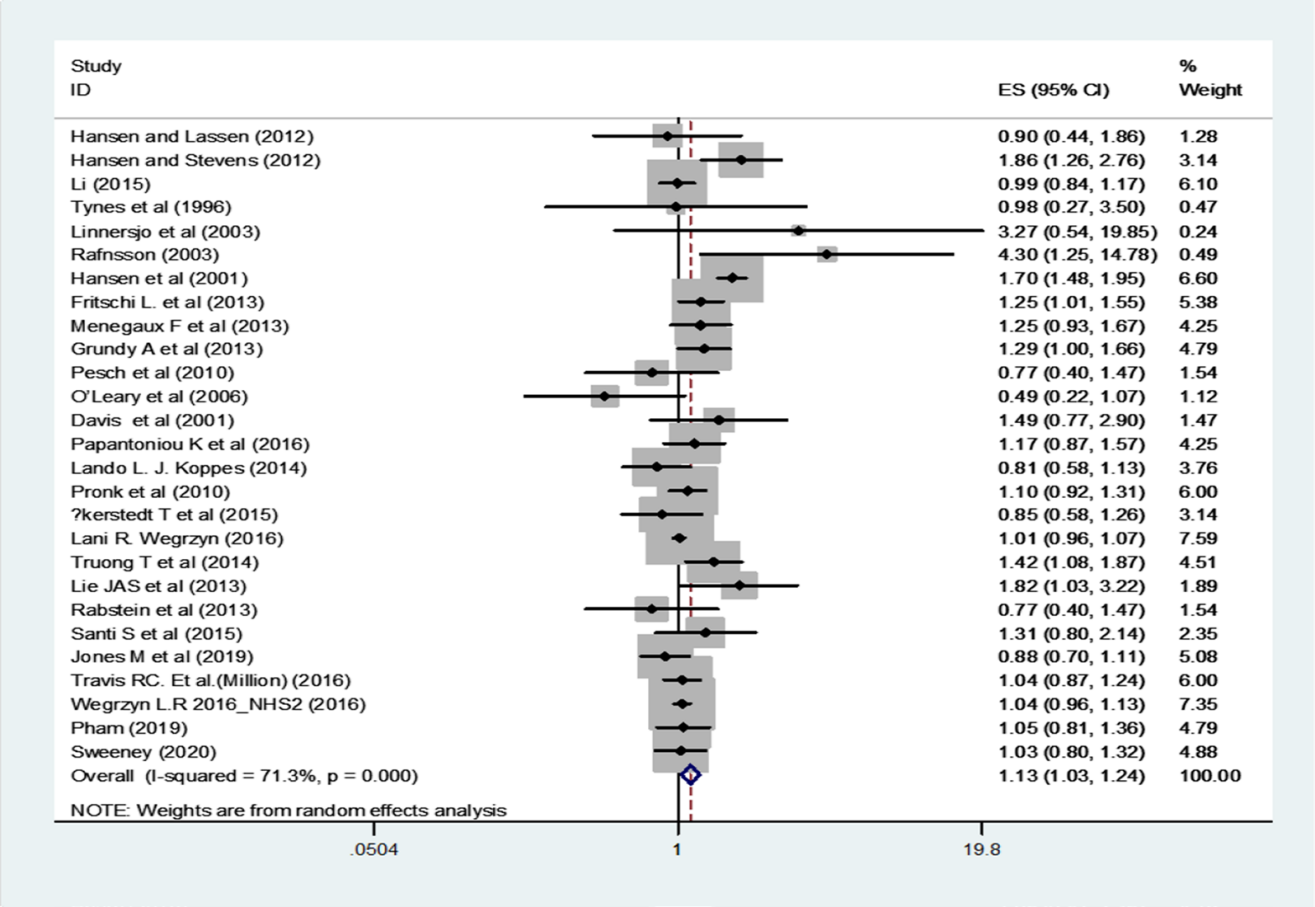
Forest plot showing risk of breast cancer for short-term (< 10 years) versus never night shift workers

**Fig. 3:**
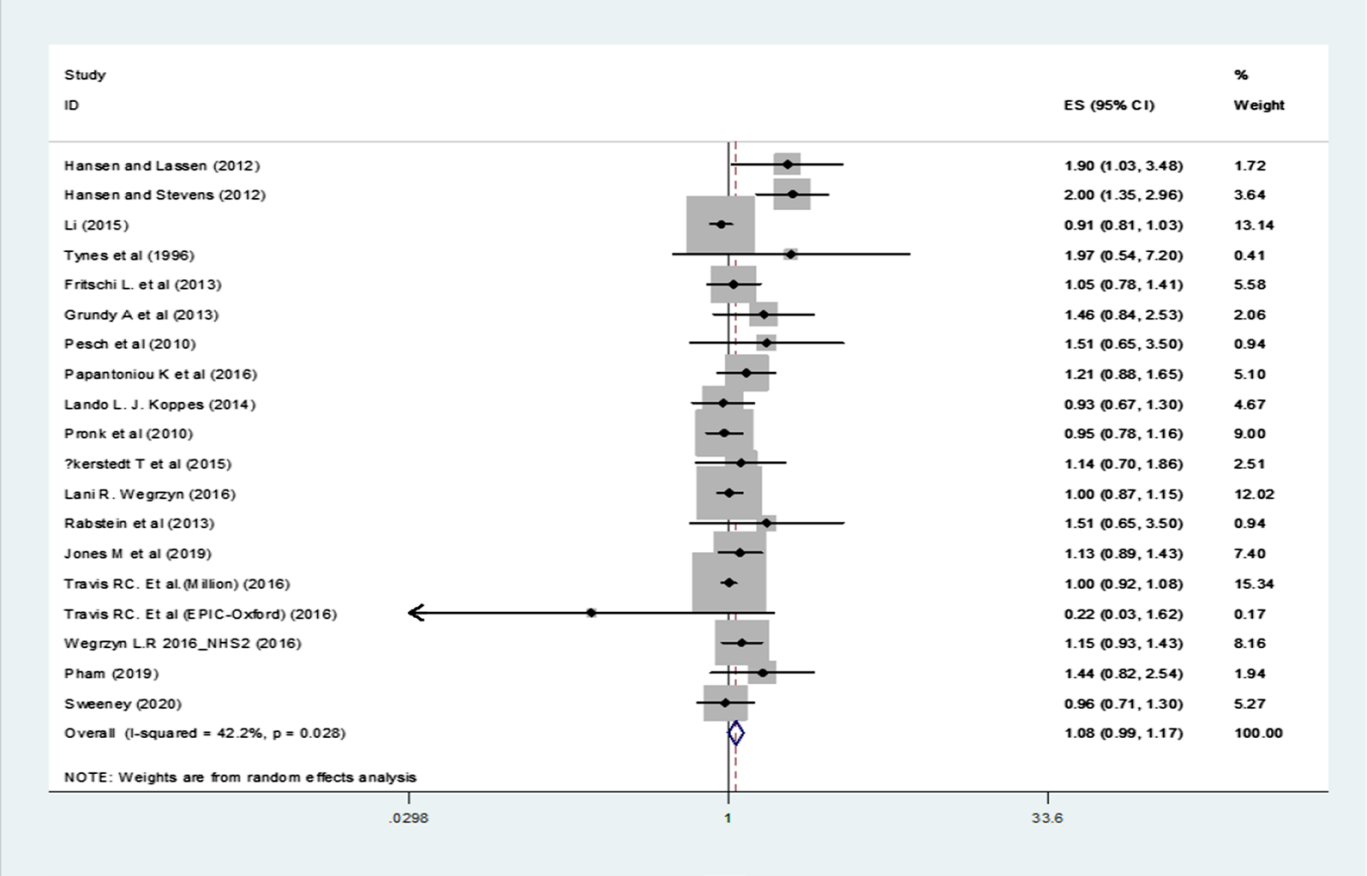
Forest plot showing risk of breast cancer for long-term (≥ 10 years) versus never night shift workers

In the cases of short-term NSW (< 10 years), BC risk was significantly increased (RR = 1.13, 95% CI 1.03–1.24, p = 0.008, I^2^ = 71.3% and p < 0.001) (Fig. 2; Table 4) but the increase was not significant in the cases of long-term NSW (≥ 10 years) (RR = 1.08, 95% CI 0.99–1.17, p = 0.09, I^2^ = 42.2% and p = 0.03) (Fig. 3; Table 4), with moderate to significant statistical heterogeneity observed in both groups.

### Subgroup analyses

The association of NSW with BC was assessed in subgroups including study type (nested case-control, case-control or cohort), occupation (nurses, flight attendants, or others), geographical area (Europe, North America, Asia and Oceania), adjustment of studies for reproductive factors (yes or no), as well as family history of BC (yes, no) , quality category (high or moderate) and reporting the source of funding (yes or no) regarding short-term and long-term exposures separately. In some of the subgroups, a significantly increased BC risk was observed in women involved in NSW (Table 4).

#### For short-term versus never NSW the results were as follows

A statistically significant association was found between short-term NSW and risk of BC in the case–control subgroup (RR = 1.25, 95% CI 1.08–1.44). In flight attendants, the short-term NSW increased the risk of BC (RR = 3.94, 95% CI 1.42–10.91). There was no significant association identified between short-term NSW and risk of BC in North America (Table 4). The subgroup analysis showed a significant association between short-term NSW and BC risk in the subgroups adjusted for the status of reproductive (RR = 1.15, 95% CI 1.05–1.27) and family history (RR = 1.11, 95% CI 1.03–1.2). A significant relationship was further observed between short-term NSW and BC risk in high quality studies (RR = 1.15, 95% CI 1.04–1.26) (Table 4).

#### In regard to long-term versus never NSW, the results were as follows

There was a significant association between long-term NSW and BC risk in case–control studies (RR = 1.22, 95% CI 1.02–1.46). Stratification of the studies by occupation revealed that there was no significant relationship between long-term NSW and risk of BC (RR = 1.03, 95% CI 0.95–1.11). As far as the geographic area is concerned, the association between long-term NSW and risk of BC was significant only in Europe (RR = 1.21, 95% CI 1.02–1.44) (Table 4). Also, there was a significant association between long-term night-shift work and BC risk in the subgroups adjusted for the status of reproductive factors (RR = 1.1, 95% CI 1–1.21) and positive family history of BC (RR = 1.1, 95% CI 1–1.2). A significant relationship was also observed between short-term night-shift work and BC risk in high quality studies (RR = 1.1, 95% CI 1–1.21) (Table 4).

### Heterogeneity analysis

To evaluate heterogeneity between included studies the X^2^ and I^2^ statistics were used. Overall, the heterogeneity in the short-term NSW was high (I^2^=73.4%). Also, heterogeneity was observed in six subgroup analyses (i.e., case-control studies, other occupations, adjustment for reproductive factors and confounders, high quality studies and, low risk studies for source of funding) (Table 4).

The heterogeneity in the long-term NSW was low (I^2^=42.2%). In the long-term NSW group, the heterogeneity was removed in case-control studies, cohort studies and, the studies from North America (I^2^=0.0%). In this group, heterogeneity was observed in three subgroup analyses (i.e., nested case-control studies, nurses and, European countries.)

### Publication bias

Egger and Begg tests and the funnel plot for short-term (Additional file 1: Appendix) did not provide significant evidence on the publication bias in the short NSW exposure group (Egger: p = 0.56; Begg: p = 0.35). However, Egger and Begs tests (Egger: p = 0.003; Begg: p = 0.09) and the funnel plot for long-term (Additional file 1: Appendix) provided evidence for the publication bias. For further assessment on publication bias in long-term night-shift workers, we ran trim and fill method [77, 78]. This technique enables us to investigate the potential effect of publication bias. It employs an algorithm to impute potentially missing studies for the reason of publication bias and generates a funnel plot that includes both the observed studies and the imputed studies, so that when the imputed studies are included, the researcher can see how the effect size changes. This approach does not require any assumptions about the process leading to publication bias, provides an estimation of the number of missing studies, and also, based on the filled studies, provides an 'adjusted' estimated impact for the publication bias [77, 78]. In this imputation method on our study, five hypothetically missing studies were imputed, as square shapes, in funnel plot (Additional file 1: Appendix). Based on the results of trim and fill imputation method the ‘adjusted’ point estimate is almost close to the (RR = 1.02, 95% CI 0.91–1.15). It is worth noting that despite the lack of publication bias in short-term night-shift workers, we ran trim and fill method, and obviously no missing study was imputed (Additional file 1: Appendix).

## Discussion

Using a comprehensive literature review, this systematic review and meta-analysis presented a significant update on the relationship between NSW duration and risk of BC. Based on the overall evaluation of the twenty six articles, in the short-term night-shift workers, the risk of BC was increased (RR = 1.13, 95% CI: 1.03–1.24), but the increase did not observe in the long-term night-shift workers (RR = 1.08, 95% CI 0.99–1.17).

### Previous meta-analysis of NSW and BC

The first meta-analysis in this regard was reported in 2005 on six studies, revealing an increase in the risk of BC among night workers (RR = 1.51; 95%, CI: 1.36–1.68) [18]. Erren et al. [31] concluded that the risk of BC in the shift workers increases by 40%. Following IARC evaluation in 2007, four meta-analyses on BC and NSW were published in 2013 [24, 26, 29, 79]. The results of these studies were contradictory in regards to the effect of NSW on BC. Jia et al. found an overall positive correlation of 1.20 (95% CI 1.08–1.33; 13 studies) between the risk of BC and NSW (never versus ever) [26], which is also consistent with the study of Wang et al. [24] and Ijaz et al. [79]. But another review in the same year [29] obtained contradictory results and found no significant association between NSW with increased risk of BC.

Ijaz et al. [79] observed a 9% risk increase per five years of NSW exposure in case–control studies (RR = 1.09, 95% CI: 1.02–1.20), a finding that was not reported in cohort studies [6, 39, 46, 49, 74]. Ijaz et al., due to the low incidence of BC, took both odds ratios (OR) and risk ratios (RR) as valid estimates of the relative risk. So, they reported their results as RR, which can be seen in the original article. Travis et al. [28] concluded that NSW, including long-term NSW, has no effect on BC incidence, which is in line with the results of the current study. But He et al. [25] and in later years Yuan et al. [27] reported a significantly positive association between NSW and BC risk. A recent meta-analysis by Dun et al. did not find an overall association between NSW and the risk of BC [30].

However, in Wang et al. study [24], the meta-regression showed a rise of BC risk with the duration of NSW and cumulative night work (pooled RR = 1.03, 95% CI 1.01–1.05; Pheterogeneity < 0.001). In accordance with Wang et al., Yuan et al. mete-analysis showed that the risk of BC is higher in long-term night-shift workers (OR = 1.316; 95% CI1.196–1.448) [27]. These divergent results might be attributed to the different articles included in these meta-analyses. Yuan et al. [27] in their meta-analysis incorporated some articles regarding the effect of LAN and/or sleep disruption on BC. However, the systematic review carried out by Kolstad [19], stated inadequate evidence to support the association between NSW and BC, which is inconsistent with the findings of Hansen et al. [80] and Lee et al. [81]. As observed, the results of previous meta-analyses are quite contradictory. According to the results of the present meta-analysis, the risk of BC increases in short-term NSW, while this increase is not obvious in the long-term NSW group. This result differs from studies that reported an increased risk of BC as the years of NSW increased [24, 27, 79]. It seems that this inconsistency is due to the fact that some meta-analyses have considered ever vs. never NSW, but in the present study, the duration of NSW has been considered.

According to the results of the subgroup meta-analysis, employment in NSW (as short term and long term) do not increase the risk of BC risk. According to our results and the meta-analyses by Dun et al. [30] and Travis et al. [28], among the groups of nurses, NSW does not increase the risk of BC, which are inconsistent with some previous studies [24, 25, 27]. Significant heterogeneity was observed in our results and some other meta-analyses [25, 27, 29, 79]. Generally, the heterogeneity observed in the findings of epidemiological studies can be partially assigned to the large differences in the NSW definition, design of the study, duration of the follow-up period, left-truncation in cohort studies, lack of chronotype information, social jet lag, and differences in the menopausal status of the population under study as well as subtypes of BC.

Similar to the findings of previous meta-analyses, we also found in the subgroup analysis that the risk of BC increases in flight attendants, with long or overnight flights [18, 25, 29, 31]. However, the role of cosmic radiation as a confounder should not be ignored in this occupational group. Erren et al. suggested a 70% increase in the risk of BC in flight attendants [31]. Due to the dearth of studies in relation to this occupational group, further research is needed for more accurate and robust results.

If the analyses were stratified by the study design, the risk of BC was increased in case–control studies but no increase was seen in cohort studies which is in line with the results reported by Dun et al. [30], He et al. [25] and Ijaz et al. [79]. But our results are inconsistent with the findings of a pooled analysis of case–control studies proposed that BC risk did not increase with the lifetime duration of night work or with the duration of night shifts in both pre-and post-menopausal women; moreover, the risk might decrease after the cessation of exposure [82]. As a common concern in case–control studies, during the evaluation of night work, recall bias may have been incorporated into our research. This bias is a significant challenge to the validity of self-reported questionnaires when the participants were examined. To remove possible recall bias arising from previous case–control studies on the relationship between NSW and BC, We examined the findings of cohort studies in which effective control of recall bias was possible. There was not an insignificant relationship again. Our subgroup analysis revealed that NSW is related with increasing BC risk in European countries that is in line with some other meta-analyses [25, 30]. The most important risk of bias in the studies included in the meta-analysis was measurement of exposure which is reported in other studies [79].

The lack of association between long-term NSW and BC could be due to the healthy worker effect. A healthy worker effect is a special form of selection bias common to occupational cohort studies that occurs because healthy individuals are less likely to be unemployed than are unhealthy individuals [83, 84]. The main mechanisms for the healthy worker effect in this study are health-based differential losses to follow up (healthy worker survivor effect), health-based selection of workers in long-term NSW (healthy hire effect) [84].

### Strengths and limitations

One of the strengths of our systematic review and meta-analysis was that in the present study, due to an updated literature search, some recent publications, that were not included in previous meta-analyses, were reviewed [28, 69, 71, 75]. From those one study published in 2019 [71] was a large cohort study, and the other reported the findings of three large cohort studies in England [28], which included two cohort reports (one of them only reported "ever" versus "never" NSW). Therefore, we included three cohort studies in two articles. We also incorporated seven studies published from 2013 to 2020 from different countries, one study on nurses [73] with 24 years of follow up (which reported two cohort study: NHS and NHS2), a large cohort study (sister study) that has published recently [75] and four population-based case–control studies [22, 62, 64, 69]; to our knowledge, this is the first time that such studies are included in a meta-analysis based on the duration of NSW. Although the generalizability of our results was enhanced by studies involving larger and more diverse populations, but they adversely increased between-study heterogeneity, which resulted in pooled RRs that were not consistent with previous studies, and more difficult to interpret. Second, several subgroup analyzes were performed to discover whether stratification by study type, occupation, geography, and study design (adequate adjustment for confounders, reproductive factors and family history of BC), article's quality category and to report the source of funding are able to minimize the heterogeneity of pooled analyses and suggest expressive associations for the current and future research. Some of the previous meta-analyses incorporated articles concerning the effect of LAN and/or sleep disturbances on BC [25, 27]. In these meta-analyses [25, 27], no classifications were done based on the duration of NSW; however, in the present study, the NSW was classified into two categories: short-term and long-term night-shifts. Third, we used several methods (funnel plots, Begg's and Egger's test, trim and fill test) to investigate the publication bias in short-term and long-term exposure groups, separately. The previous meta-analyses did not include the risk of bias assessment [85].

Our study had several limitations. Firstly, considerable variability of study design,, study population, sample size, definition of NSW, mode of exposure quantification, risk estimates, and adjustment for pertinent confounders, may restrict the generalizability of our findings to specific populations. Secondly, our included studies showed various rates of bias, specifically the recall bias associated with self-reported exposures, as seen in many observational studies. Certain included studies (69%) were designed as (nested) case–control, hence particularly susceptible to recall bias, which can lead to heterogeneity and contradictory results between papers. However, in every study, the authors apply different methods in order to reduce the recall bias, as mentioned previously.. Finally, we might have missed some studies in local languages.

Accordingly, in future studies, exposure must be measured with an objective scale in cohorts with long follow up. In addition, for common confounders, not all studies are obtained or adjusted, further reducing the strength of the exposure-outcome association. It is also recommended that authors focus on the quality of reporting of different sections of articles (especially exposure/outcome details) and report their articles on the basis of quality assessment tools.

## Conclusion

The present meta-analysis showed a positive statistical relationship between NSW and BC risk in short-term night-shift workers but no increase was observed in the long-term night-shift workers. Night-shift workers including flight attendants were associated with increased BC risk. Our subgroup analysis revealed that flight attendants with long overnight flights were at an elevated risk of BC. In this case, however, more studies are needed for more robust results. Also, according to the results of the subgroup analysis, the risk of BC in case–control studies adjusted for reproductive factors and family history of BC, as well as studies with high quality were increased in both short term and long term NSW. We recommend that, BC screening services should be integrated to the routine care for women with night-shift jobs. We suggest further studies with adequate information and exact definition regarding NSW and its duration.

## Supplementary Information

**Additional file 1**. Funnel plots of the articles performing short and long-term versus never night-work—shift work analysis—Trim and fill test for short and long-term night-shift work—Begg's and Egger's test for publication bias—Risk of bias graph—Quality assessment of included studies—PRISMA checklist

## Data Availability

The datasets used and/or analyzed during the current study are available from the corresponding author on reasonable request.

## Abbreviations

IARC: International Agency for Research on Cancer
BC: Breast cancer
NSW: Night-shift work
NOS: Newcastle–Ottawa Scale
LAN: Light at night
